# Protective effect of phosphoenolpyruvate carboxykinase 1 on inflammation and fibrotic progression of IgA nephropathy

**DOI:** 10.1080/0886022X.2025.2508297

**Published:** 2025-05-29

**Authors:** Ya-yin Tan, Yu-kai Wang, Xiao-mei Luo, Chao-yi Chen, Xue-qi Liu, Xin-ran Liu, Yong-gui Wu

**Affiliations:** Department of Nephropathy, The First Affiliated Hospital of Anhui Medical University, Hefei, Anhui, PR China

**Keywords:** IgA nephropathy, PCK1, PPARγ, inflammation, fibrosis

## Abstract

**Introduction:**

Phosphoenolpyruvate carboxykinase 1 (PCK1) is an essential enzyme of the gluconeogenic pathway, which can affect kidney physiology in various ways. Nevertheless, its role in the progression of IgA nephropathy (IgAN) remains to be elucidated.

**Methods:**

We identified the differentially expressed genes in the glomeruli of IgAN patients through weighted gene co-expression network analysis across three datasets. Through clinical renal pathological tissue and cellular experiments, we further validated the gene and investigated its relationship with the inflammatory markers and fibrosis indicators in IgAN.

**Results:**

Compared to peritumoral normal tissues, the peroxisome proliferator-activated receptor γ (PPARγ) signaling pathway have been identified as key elements in IgAN pathogenesis from two GEO databases and a validation dataset. PCK1 was identified and validated as one of the most promising candidate genes. The expression of PCK1 in clinically collected kidney specimens was significantly downregulated in patients with IgAN compared to healthy controls. The expression of PCK1 was inversely correlated with clinical indicators such as urinary albumin-to-creatinine ratio, 24-hour proteinuria. In experiments with SV40-transformed mouse glomerular mesangial cells (MCs), PCK1 and PPARγ protein expression levels were significantly decreased in polymeric IgA1 (pIgA1)-stimulated MCs, which contrasts with the increased expression of inflammatory and fibrotic factors. Overexpression of PCK1 inhibited cellular inflammation and fibrotic changes induced by pIgA1, demonstrating protective effects against cellular fibrosis similar to rosiglitazone.

**Conclusion:**

PCK1 exerted a pronounced inhibitory effect on mesangial cell inflammatory markers and fibrosis indicators in IgAN, potentially offering a novel therapeutic target for its treatment.

## Introduction

IgA nephropathy (IgAN) is increasingly acknowledged as the predominant primary glomerular disorder associated with the development of chronic kidney disease (CKD). Renal biopsies confirm IgAN in approximately 30–40% of cases, with a frequency ranging from 29% to 53% in Asia, surpassing observed rates in Europe [1]. The disease progresses insidiously, often causing individuals to mistakenly perceive a favorable prognosis. However, epidemiological data demonstrate that within a decade following the onset of clinical symptoms, approximately 15–20% of patients will progress to end-stage renal disease (ESRD) [2,3], with some reports suggesting this proportion may even reach approximately 40% [4,5]. The primary cause of IgAN is widely recognized to be the deposition of immunoglobulin in the mesangial area of the glomerulus; nevertheless, the precise pathogenic mechanisms remain incompletely understood [6].

Current studies on the pathogenesis of IgAN largely support the ‘quadruple hit’ theory [7]. According to this theory, the terminal event occurs within the glomerulus, where polymeric IgA1 immune complexes with defective O-glycosylation bind to transferrin receptor (TfR) on glomerular MCs, thereby activating these cells. This activation leads to abnormal MCs proliferation, excessive extracellular matrix synthesis, and the release of inflammatory cytokines and chemokines that disrupt podocyte and renal tubular cell function. Therefore, MCs are considered key intrinsic cells in IgAN pathogenesis. Proliferation of glomerular mesangial cells and accumulation of extracellular matrix represent fundamental pathological features of IgAN [8].

Renal biopsy remains an essential diagnostic tool for IgAN. Blood pressure, proteinuria, and renal function are key determinants of prognosis, while the incorporation of MEST-C and KATAFUCHI histological scoring systems can enhance prognostic accuracy [9]. General measures for kidney protection, including lifestyle modifications, blood pressure management, control of protein and salt intake, and inhibition of the renin-angiotensin-aldosterone system (RAASi), form the cornerstone of IgAN treatment [10]. However, the treatment of diseases requires more precise and less toxic immunosuppressive methods, especially those targeting specific molecular pathways.

Phosphoenolpyruvate carboxykinase 1 (PCK1) is a cytoplasmic enzyme that, in conjunction with pyruvate carboxylase during gluconeogenesis, catalyzes the conversion of oxaloacetate to phosphoenolpyruvate. It also plays a crucial role in the PPAR-γ signaling pathway. In mammals, two isoforms of PCK have been identified: the cytoplasmic isoform encoded by the PCK1 gene and the mitochondrial isoform encoded by the PCK2 gene. Notably, PCK1 is predominantly expressed in human kidney tissues [11,12]. Research has demonstrated that beyond its function as a rate-limiting enzyme in gluconeogenesis, PCK1 is extensively involved in various metabolic and biological processes, including glucose and lipid metabolism, aging, inflammation, fibrosis, and tumor cell proliferation and apoptosis [13–18]. Recent studies have highlighted the significant role of PCK1 in the progression of diabetic nephropathy and acute tubular injury [19,20].

However, the alterations in PCK1 and the PPAR-γ pathway in IgAN, as well as the impact of PCK1 on inflammation and fibrosis in this condition, remain unclear. To address these issues, we investigated the regulatory role of PCK1 by analyzing clinical renal biopsy specimens from patients and utilizing an *in vitro* IgAN model of SV40-transformed mesangial cells (MCs). Through these experiments, we aim to elucidate changes in PCK1 expression in IgAN and its effects on inflammation and fibrosis in MCs, with the goal of determining whether PCK1 can serve as a potential therapeutic target for the disease.

## Materials and methods

### Screening for co-DEGs

**Figure 1.**
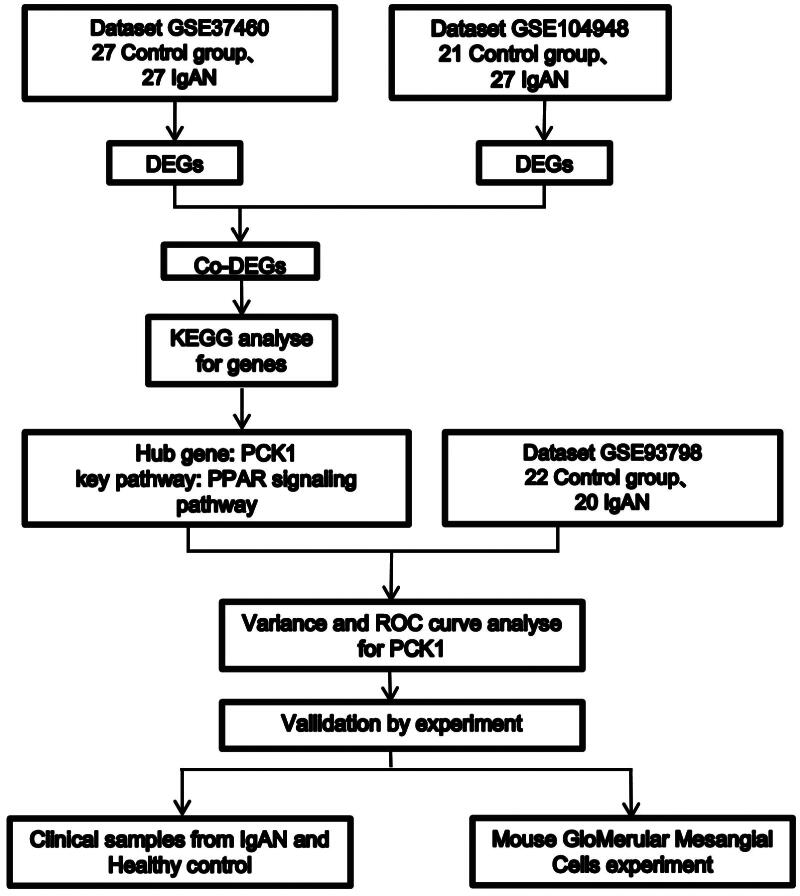
Flow diagram illustrating the study design and workflow.

In the GEO database (http://www.ncbi.nlm.nih.gov/geo/), a search for ‘IgA Nephropathy’ with the species set to ‘Homo sapiens’ and the study type refined to ‘Expression profiling by array’ was performed to obtain relevant gene expression profiles. The inclusion criteria for datasets were: (1) data from expression profiling microarray analysis; (2) use of microdissected glomerular samples; and (3) no duplicate sample data within each dataset. Consequently, the datasets GSE93798 [21], GSE37460 [22], and GSE104948 [23] were meticulously selected for this study, as illustrated in Figure 1. Initially, datasets GSE37460 and GSE104948, containing renal tissue data from patients with IgAN and healthy controls, standardized by the ‘Limma’ package in R, and visualized with the ‘ggplot2’ package. were screened to identify DEGs. Probe-to-gene conversion was conducted using the respective platform’s annotation files, with the gene expression value determined as the average of the probes. The selection cutoff was adj.*p* < 0.05 and |log2FC| > 1.2, identifying significantly differentially expressed genes (DEGs). The DEGs were visualized using heatmaps and volcano plots, while a Venn diagram was employed to illustrate the overlapping DEGs between the two datasets. Utilize the ‘ggplot2’ package to generate boxplots depicting the differential expression of the PCK1 gene across various datasets, employing the Wilcoxon rank-sum test for statistical analysis. Additionally, leverage the ‘pROC’ package to assess the impact of the target gene PCK1 on the classification and diagnosis of IgA nephropathy compared to the control group.

Kyoto Encyclopedia of Genes and Genomes (KEGG) analysis on the shared DEGs from both datasets was performed to pinpoint hub genes and key pathways.

Using the dataset GSE93798 as the validation set, the expression differences of the hub gene were plotted using the R package ‘ggpubr’ and displayed as box plots. The significance between groups was calculated using the Wilcoxon test. Additionally, the ‘pROC’ package in R was employed to validate the diagnostic accuracy of this key gene for diseases in the validation set, with ROC curves plotted for the key gene.

Based on the target gene PCK1, we employed the STRING database (version 12.0) to identify proteins interacting with the encoded protein of this gene and constructed a protein-protein interaction (PPI) network. We subsequently conducted KEGG and GO enrichment analyses on the identified interacting proteins using the R package ‘clusterProfiler’. For p-value correction, we applied the Benjamini-Hochberg (BH) method, while all other parameters were retained at their default settings.

### Antibodies and reagents

PPAR-γ antibody (16643-1-AP), PCK1 antibody (16754-1-AP), and fibronectin polyclonal antibody (15613-1-AP) were sourced from Proteintech Group (Wuhan, China). Anti-collagen IV antibody (bs-4595R) and rabbit Anti-alpha smooth muscle Actin antibody (bs-10196R) were obtained from Bioss Antibodies (Beijing, China). Anti-TGF beta antibody (ARG10002), anti-TNF-α (ARG10158), and anti-IL-1β (ARG66285) were procured from Arigo biolaboratories (Shanghai, China). Horseradish peroxidase-conjugated goat antibodies against mouse IgG (ab6789) and rabbit IgG (ab205718), as well as anti-smad3 antibody (ab40854), anti-Phospho-smad3 (ab138659), anti-beta Actin antibody (ab8226), and anti-PDGFR beta antibody (ab313777), were provided by Abcam (Cambridge, MA, USA). Hematoxylin and eosin staining (H&E) kits, periodic acid-Schiff (PAS) kits, and periodic acid-silver methenamine (PASM) kits were procured from Beijing Solarbio Technology Co., Ltd. (Beijing, China). The monomeric human IgA1 (mIgA1) were procured from Abcam (ab91020).

### Collection of renal tissues

This study received approval from the First Affiliated Hospital of Anhui Medical University (approval no. PJ 2024-01-24). Hospitalized patients diagnosed with primary IgA nephropathy through renal biopsy between August 2022 and October 2023 were enrolled. Paraffin-embedded pathological tissues were obtained from kidney biopsies. Additionally, patients diagnosed with renal cancer were recruited, and kidney tissue samples located 5 cm away from the tumor site were collected as control specimens. These samples were confirmed by pathology experts to be histologically normal. The recruitment criteria for the healthy control group included: a glomerular filtration rate exceeding 90 mL/min/1.73 m^2^, normal proteinuria (urinary albumin less than 30 mg/d or 20 mg/L), normal blood glucose levels, optimal liver function, and a complete blood count. The IgAN group required a pathological diagnosis of IgA nephropathy and exclusion of secondary diseases. Pathological typing was conducted using the 2017 Oxford classification (MEST-C). Participants must have no history of hormone or immunosuppressant usage prior to enrollment. Exclusion criteria included hepatitis B-related, metabolic-related, autoimmune-related, tumor-related, or other secondary forms of IgAN; liver dysfunction; pregnancy or lactation; existing infections or stress conditions; and severe cardiovascular diseases. A total of 30 healthy control subjects and 79 patients diagnosed with IgA nephropathy who met the inclusion criteria were enrolled. The primary laboratory results and pathological information used for patient recruitment are presented in Supplementary Tables 1 and 2.

### Pathological staining, immunofluorescence, and immunohistochemistry testing of glomeruli

Histological examination (HE), periodic acid-Schiff (PAS), Masson’s trichrome, periodic acid-Schiff-reticulin (PASM) staining, and immunofluorescence labeling were performed on a diverse array of tissue sections. Renal tissues from both groups underwent immunofluorescent double staining to localize platelet-derived growth factor receptor-beta (PDGFR-β) and PCK1. The paraffin sections of the kidney were dewaxed, and antigen retrieval was performed using citrate solution. The sections were incubated with hydrogen peroxide for 20 min, followed by blocking with goat serum. The primary antibody was then added and incubated overnight in a 4 °C wet box refrigerator. After washing the sections, the secondary antibody was incubated at 37 °C for 30 min. The sections were stained with 3,3′-diaminobenzidine (DAB). Hematoxylin was used to stain the nuclei. The sections were observed using a Leica microscope (Leica, Bensheim, Germany). The glomerular region was examined for protein expression using Image-Pro Plus v6.0, with three randomly selected positive stained areas evaluated by two pathologists through semi-quantitative analysis.

### Electron microscopy

The tissue was fixed with glutaraldehyde (2.5%) and osmium tetroxide (1%) at 4 °C for 3 days. Samples were then treated with solution of uranyl acetate (1%) and then embedded in epoxy resin (EPON). Polymerization occurred in gelatin capsules at a temperature of 60 °C for a duration of 48 h. Subsequently, the resulting slices were examined using a transmission electron microscope (Hitachi, Japan).

### Cell culture

The SV40 MES 13 cells used in this study were obtained from the cell repository of the Chinese Academy of Sciences. They were cultured in a medium consisting of Dulbecco’s Modified Eagle Medium (DMEM) supplemented with 10% fetal bovine serum (FBS).

### Stimulation of MCs with pIgA1

The mIgA1 was subjected to a thermal treatment at 65 °C for a duration of 150 min to generate polymeric human IgA1 (pIgA). MCs were then stimulated with pIgA to establish an Immunoglobulin A nephropathy (IgAN) disease model [24–26].

### MTT assay

The MTT cytotoxicity and cell proliferation detection kit was obtained from Beyotime Biotechnology (Shanghai, China). MCs were stimulated with different concentrations of pIgA1 (0 μg/mL, 12.5 μg/mL, 25 μg/mL, 50 μg/mL, and 100 μg/mL). Each experimental group consisted of six replicate wells. MTT solution was introduced into every well, and the reaction was halted after a 24-h incubation period. The absorbance at 550 nm was measured using an ELISA reader to quantify the results.

### Transfection with PCK1 overexpression lentivirus

The PCK1 lentivirus transfected plasmid (PHBLV002408) was constructed by Hanbio (Shanghai, China). Supplementary Table 3 lists all the primer sequences used in this investigation. Two centrifuge tubes were prepared for the empty vector and overexpression PCK1 plasmids, respectively. Each tube received 200 μL Opti-MEM and 5 μL siRNA/Lipofectamine 2000 (Invitrogen, USA). After standing for 5 min, mix the two tubes together and then let it stand for additional 20 min. The cells were subjected to starvation treatment in a prepared culture medium. Subsequently, 600 μL of Opti-MEM (Gibco, USA) was added to each well. Then, 400 μL of the pre-mixed solution was gently added to each well, ensuring thorough mixing. The plate was incubated for 6 h. Post-transfection, the medium was substituted with a 5% FBS-containing solution, and the cells were cultured for 24 h to facilitate mRNA extraction for qRT-PCR analysis. For protein extraction and subsequent Western blotting, a 48-h cultivation period was required.

### Cell stimulation by rosiglitazone

The cells were subjected to a 48-h stimulation with rosiglitazone (Yindole Biotechnology Co., Ltd, Shanghai, China), dissolved in DMSO at a concentration of 5 mg/mL, and then added to RPMI to achieve a final concentration of 10 μmol/L and 0.01% (vol./vol.) DMSO. The dosage of rosiglitazone was determined in accordance with the findings from previously published *in vitro* studies [27–30].

### Western blotting analysis

Protein quantification in renal tissues or cells was performed using a BCA protein kit (Beyotime) following extraction with RIPA buffer (Beyotime, Jiangsu, China). The proteins were separated by SDS-PAGE using gels of varying percentages and subsequently transferred onto nitrocellulose membranes. Subsequently, after incubating these membranes with specific antibodies, the band intensity was measured using the Amersham Imager 600 system (GE, USA), and the images were quantitatively analyzed using Image J software (NIH Bethesda).

### Extraction of RNA and analysis using real-time PCR

Total RNA was isolated from MCs in culture using the RNA-iso reagent (Takara). The concentration of RNA was determined using a NanoDrop 2000 spectrophotometer (Thermo Fisher Scientific, MA, USA), and cDNA synthesis was performed through reverse transcription utilizing RealMasterMix (Takara, Japan). PCR amplification was performed for 40 cycles using the following parameters: initial denaturation at 95 °C for 20 s, followed by annealing at 58 °C for 20 s and extension at 72 °C for another 20 s. The β-actin gene was utilized as an internal reference to standardize mRNA expression levels. Supplementary Table 4 lists all the primer sequences used in this investigation.

### Statistical analysis

The statistical analysis was conducted using the software package IBM SPSS 26.0. Semi-quantitative analysis of protein expression in IHC images was carried out using ImageJ software (version 2.3.0). The normality of the data was assessed using the Shapiro-Wilk test. For quantitative variables that followed a normal distribution, the mean ± standard deviation was used to present the data. T-tests were utilized for comparing two groups, while one-way ANOVA was employed for making multiple-group comparisons. For variables that do not follow a normal distribution, the median and interquartile range were used, and the Mann-Whitney U test was utilized for comparisons between two groups. The Pearson correlation coefficient and Spearman correlation coefficient were employed to analyze the associations between variables that follow a normal distribution and those that do not, respectively. Prediction models were established using binary logistic regression and Lasso regression methods, with ROC curves constructed to evaluate model performance. Data visualization was conducted using the R language or GraphPad Prism (version 8.0). The threshold for statistical significance in all analyses was established at *p* < 0.05.

## Results

### Screening for DEGs

#### Screening of DEGs from two datasets

The original expression data from the two datasets were normalized and compared to identify DEGs between patients diagnosed with IgAN and healthy individuals, enabling screening for potential biomarkers. In the GSE37460 dataset, 68 DEGs were detected, including 44 upregulated and 24 downregulated genes. While in the GSE104948 dataset, 80 DEGs were identified, including 53 upregulated and 27 downregulated genes. The levels of gene expression for all DEGs in both datasets are presented using volcano plots (Figure 2A), and then subjected to hierarchical cluster analysis. The outcomes are visualized as heatmaps (Figure 2B). A Venn diagram was generated to visually represent the shared and distinct genes in both datasets (Figure 2C). KEGG analysis demonstrated that the PPAR-γ signaling pathway was significantly enriched, with four genes exhibiting substantial changes. Notably, PCK1 displayed the most pronounced alteration, characterized by a logFC value of −1.619 (Figure 2D). A literature search revealed that PCK1 has been associated with diabetic nephropathy, acute kidney injury models, and renal clear cell carcinoma, and it is closely linked to proteinuria and extracellular matrix fibrosis in animal models [19,20,31,32]. Nevertheless, the role it plays in the progression of IgAN remains to be elucidated. Therefore, PCK1 was selected as the subsequent validation indicator for this study.

Figure 2.Screening of hub genes in bioinformatics analysis. (A) Screening DEGs from two datasets: Volcano plots of DEGs from GSE37460 (n = 54) and GSE104948 (n = 48). Blue points represent upregulated genes, red points represent downregulated genes, and gray points represent genes with no significant difference in expression (threshold, |fold-change|≥1.2 and adjusted *p* < 0.05); (B) Hierarchical cluster heatmaps of the DEGs from GSE37460 and GSE104948 (threshold, |fold-change|≥1.2 and adjusted *p* < 0.05); (C) Venn diagrams showing DEGs common to the two datasets; (D) KEGG pathways significantly enriched for the DEGs, including PPARγ signaling, are shown. PCK1 was identified as the most differentially expressed gene in the PPARγ pathway (|fold-change|=-1.619 and adjusted *p* < 0.05); (E) Box plot of initial expression for PCK1, demonstrating that the gene was significantly decreased in patients with IgAN relative to healthy controls in the GSE37460 and GSE104948 datasets (*p* < 0.001); (F) Verification of PCK1 by ROC curve analysis in the GSE37460 and GSE104948 datasets, comparing healthy control vs IgAN. (G) Box plot of initial expression for PCK1, demonstrating that the gene was significantly decreased in patients with IgAN relative to healthy controls in the GSE93798 dataset (*p* = 2.7e-11, *p* < 0.01); Verification of PCK1 by ROC curve analysis in the GSE93798 dataset, comparing healthy control vs IgAN. (H) PPI network of PCK1-related proteins retrieved from the STRING database (version 12.0). KEGG and GO enrichment analyses were performed based on the interacting proteins. PCK1 was mainly related to energy metabolism, AMPK and PPAR signaling pathways. Abbreviations: DEGs, differential genes.

**Figure:**
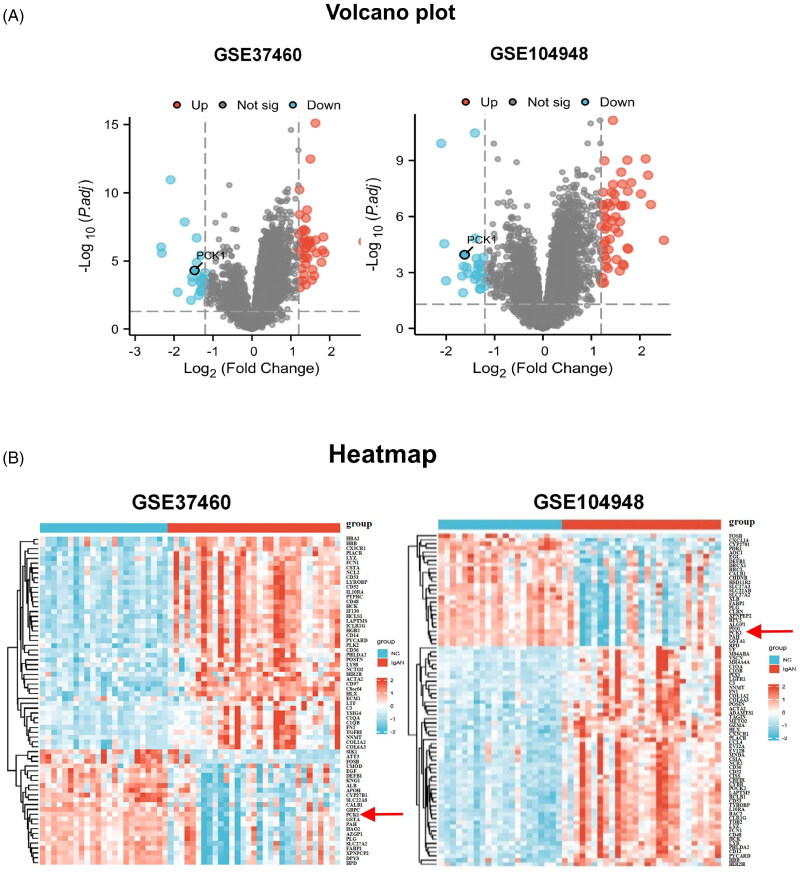

**Figure:**
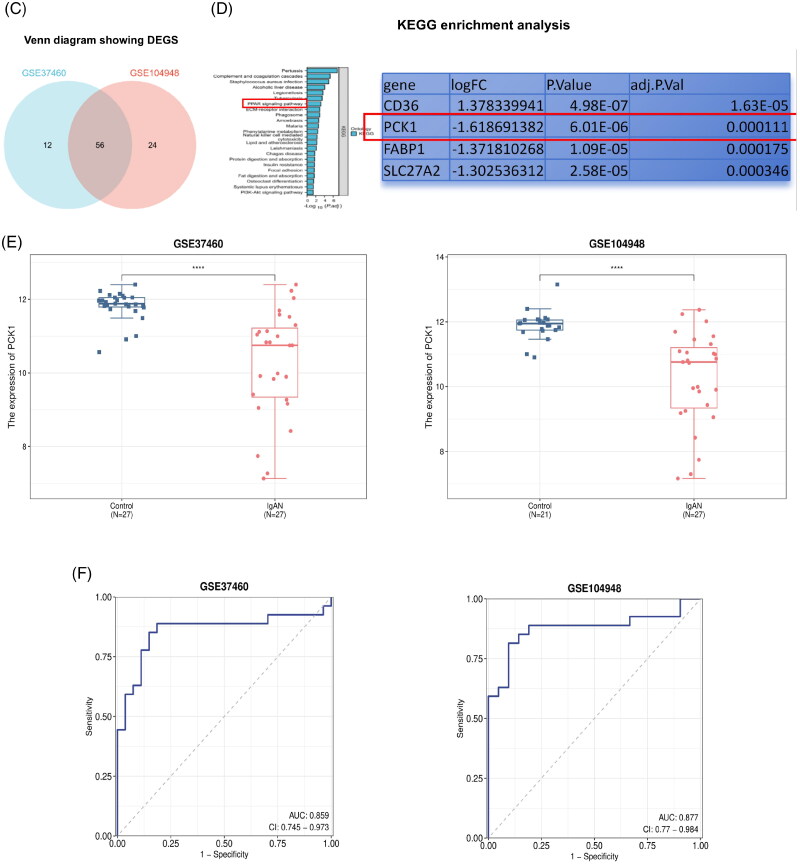

**Figure:**
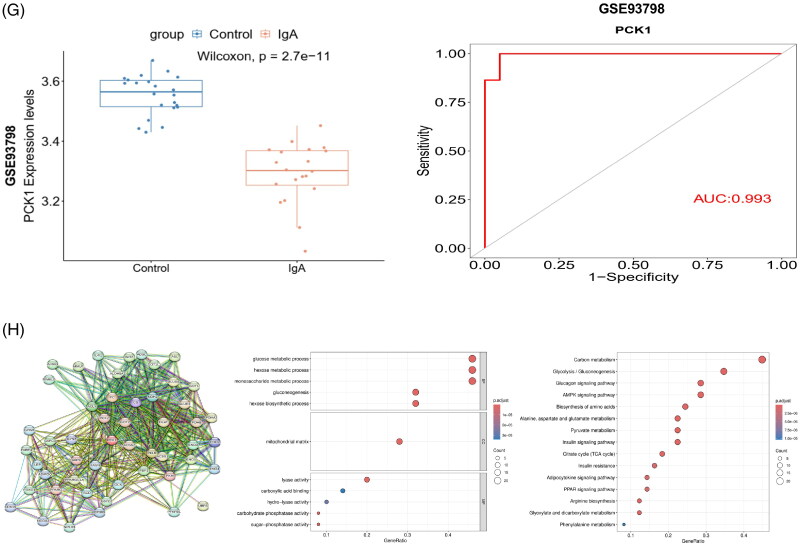

Compared with the healthy control group, the expression level of PCK1 in the disease group decreased significantly (Figure 2E). The ROC curve of the PCK1 was plotted, resulting in an AUC value of 0.859 of GSE37460 and 0.877 of GSE104948 (Figure 2F).

#### Analysis of the validation set

The GSE93798 dataset, comprising 20 IgAN glomerulus samples and 22 normal glomerulus samples, served as the validation set. Group differences were assessed using the Wilcoxon test, revealing significant downregulation of PCK1 in disease samples, consistent with the findings from the training set analysis. The ROC curve of the key gene was plotted, resulting in an AUC value of 0.993 (Figure 2G).

#### PPI network

The proteins associated with the target gene’s corresponding protein were identified using the STRING database (version 12.0), and a protein-protein interaction network was constructed. Based on the identified interacting proteins, KEGG and GO enrichment analyses were conducted using the R package ‘clusterProfiler’. The results indicate that the target gene is primarily involved in energy metabolism, AMPK signaling, and PPAR signaling pathways.

### Severe pathological changes is more pronounced in patients with IgAN

**Figure 3.**
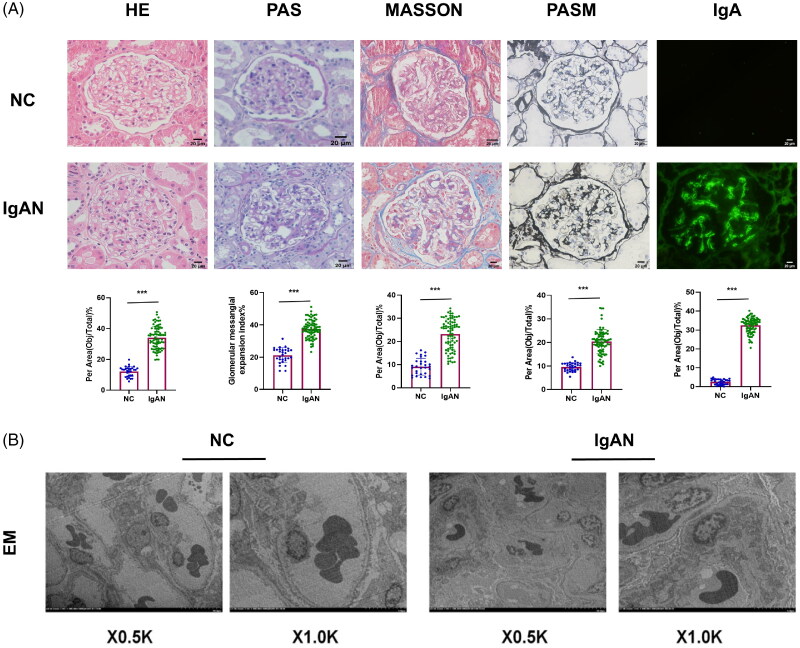
Clinical patient kidney pathology staining. (A) Pathological staining of clinical kidney specimens. Scale bar =20μm. (patients with IgAN, n = 79 and NC, n = 30). (B) Transmission electron microscopy of NC and IgAN specimens. Abbreviations: NC, normal control; H&E, hematoxylin and eosin staining; PAS, periodic acid-Schiff staining; PASM, periodic acid-silver methenamine; EM, electron microscopy. ***p < 0.001.

The NC group, as judged by the hospital’s pathology department, showed no significant pathological changes or only minor changes in staining. In contrast, HE staining revealed enhanced proliferation of glomerular mesangial cells and elevated matrix deposition in renal tissues affected by IgAN, PAS-positive deposits in the mesangial area, and basal membrane hyperplasia, among other characteristic changes, Masson’s trichrome staining indicated collagen deposition in the glomeruli, and PASM staining showed thickening of the glomerular basement membrane. Immunofluorescence staining revealed high-intensity IgA deposits in the mesangial area of renal glomeruli, often accompanied by coarse granular or clump-like deposition along the walls of capillaries (Figure 3A). Electron microscopy discerned mesangial cell and matrix proliferation, as well as granular electron-dense deposits in the mesangial region, accompanied by segmental fusion of epithelial cell foot processes, as illustrated in Figure 3B.

### Clinical patient kidney immunohistochemistry and immunofluorescence staining

#### PCK1 primarily expressed in glomerular mesangial cells (MCs)

**Figure 4.**
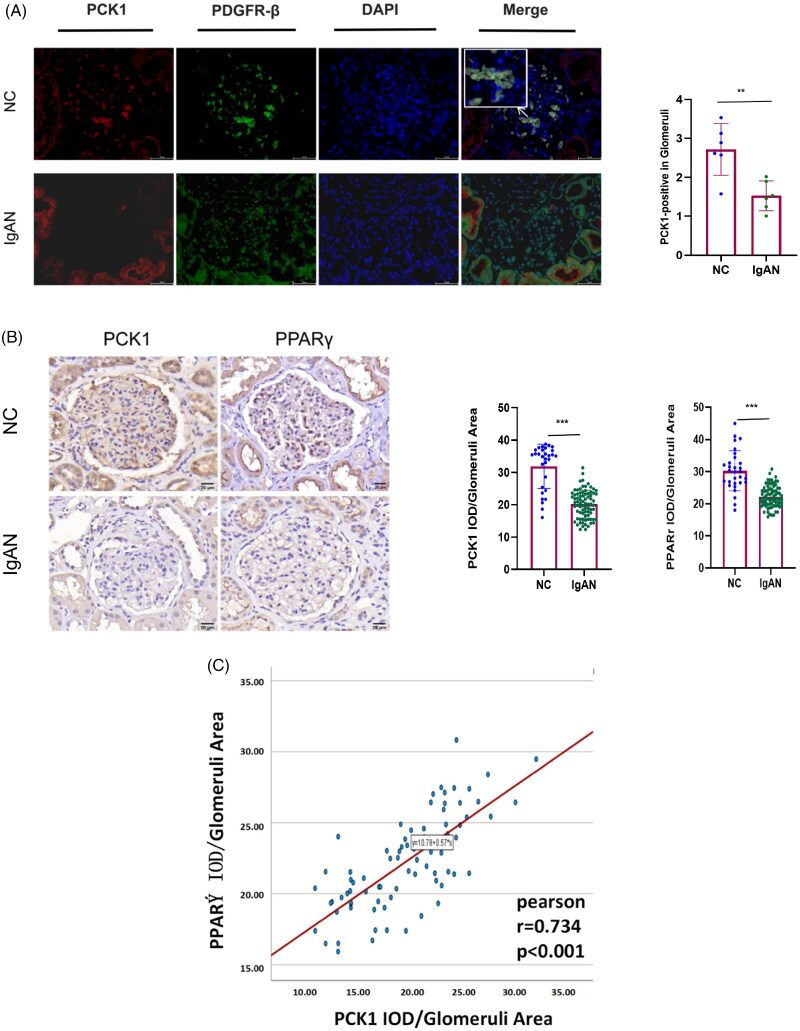
Immunofluorescence localization and immunohistochemical staining of PCK1. (A) Immunofluorescence double staining of PCK1 and the specific glomerular mesangial cells marker in renal biopsy tissues from patients with IgAN. PCK1 was labeled with a Cy3-conjugated secondary antibody, and glomerular mesangial cells were marked as PDGFR-β with a Cy3-conjugated secondary antibody; scale bar = 50μm. DAPI was used to stain cell nuclei. Merge images combine three colors. White arrows indicate characteristic staining parts, and these areas are enlarged and displayed in the upper left corner for better observation of glomerular staining results. (patients with IgAN, n = 6 and NC, n = 6). (B) Immunohistochemistry and quantification of PCK1 and PPARγ in human renal tissue (patients with IgAN, n = 79 and NC, n = 30). (C) Pearson correlation analysis of the relationship between PCK1 and PPARγ expression. PCK1 expression was linearly positively correlated with PPARγ expression (r = 0.734, *p* < 0.001). Abbreviations: NC, normal control; PDGFR-β, platelet-derived growth factor receptors beta. **p < 0.01; ***p < 0.001.

To investigate PCK1, gene expression in MCs was labeled with PDGFR-β. As shown in Figure 4A, PCK1 significantly co-localized with mesangial cell markers, with its expression notably downregulated in the diseased group.

#### Decreased PPARγ and PCK1 expression in IgAN renal tissues

For validating bioinformatics findings and exploring clinical relevance, an examination was conducted on renal tissue samples obtained from individuals diagnosed with IgAN as well as a control group consisting of unaffected subjects. Compared with the natural control group, the expressions of PCK1 and PPARγ were significantly reduced in IgAN samples (Figure 4B). Furthermore, a strong positive linear correlation was observed between the expressions of PCK1 and PPARγ (*r* = 0.734, *p* < 0.001), suggesting that the downregulation of PPARγ may contribute to the decreased expression of PCK1 (Figure 4C).

#### PCK1 expression in renal tissues shows a high correlation with pathological subtype and clinical indicators

**Figure 5.**
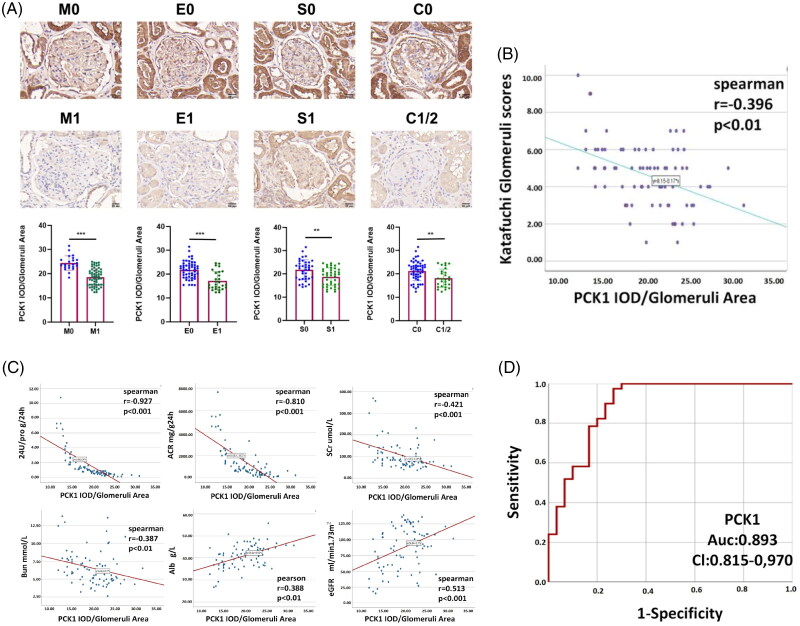
The correlation between the expression levels of PCK1 in glomeruli and glomerular pathological alterations as well as clinical parameters. (A) Immunohistochemistry and quantification of PCK1 in human renal tissue according to the Oxford classification (MEST-C). PCK1 expression in M1, E1, S1, and C1-2 categories was significantly decreased compared to M0, E0, S0, and C0 categories, respectively; (B) Spearman rank correlation analysis of the relationship between PCK1 and Katafuchi glomeruli scores. PCK1 expression was linearly negatively correlated with Katafuchi glomeruli scores (r = -0.396, *p* < 0.01); (C) Correlation scatter plot between PCK1 and clinical indicators in patients with IgAN; (D) Verification of PCK1 by ROC curve analysis. Abbreviations: AUC, area under curve; CI, confidence interval; NC, normal control; ROC, receiver operating characteristic. **p < 0.01; ***p < 0.001.

To examine the correlation between PCK1 expression in the glomeruli of patients with IgAN and the Oxford classification (MEST-C) and Katafuchi score, immunohistochemical staining for PCK1 was performed on pathological sections evaluated by a renal pathology expert. Findings showed significantly lower PCK1 expression in M1, E1, S1, and C1-2 categories compared to M0, E0, S0, and C0 (Figure 5A). Furthermore, PCK1 expression was negatively correlated with the Katafuchi semi-quantitative glomerular score (r = −0.396, *p* < 0.01), indicating that the IgAN patients with decreased PCK1 protein expression had higher Katafuchi scores (Figure 5B).

Correlation analysis indicated a negative associations were observed with 24-h proteinuria (r = −0.927, *p* < 0.001), mAlb/U-CRE (r = −0.81, *p* < 0.001), serum creatinine (r = −0.421, *p* < 0.001), and blood urea nitrogen (r = −0.387, *p* < 0.01) and positive correlation with serum albumin (*r* = 0.388, *p* < 0.001) and eGFR (*r* = 0.513, *p* < 0.001) (Figure 5C). Detailed correlation analysis between PCK1 expression and clinical features is provided in Supplementary Table 5.

An ROC curve was generated for IgAN diagnosis, with the NC group on the horizontal axis and the IgAN group on the vertical axis. Results indicated that PCK1 (AUC = 0.893, CI 0.815-0.970, *p* < 0.001) has significant diagnostic value for IgAN (Figure 5D).

#### PCK1 expression in renal tissues is associated with renal inflammatory cytokines and markers of fibrosis

**Figure 6.**
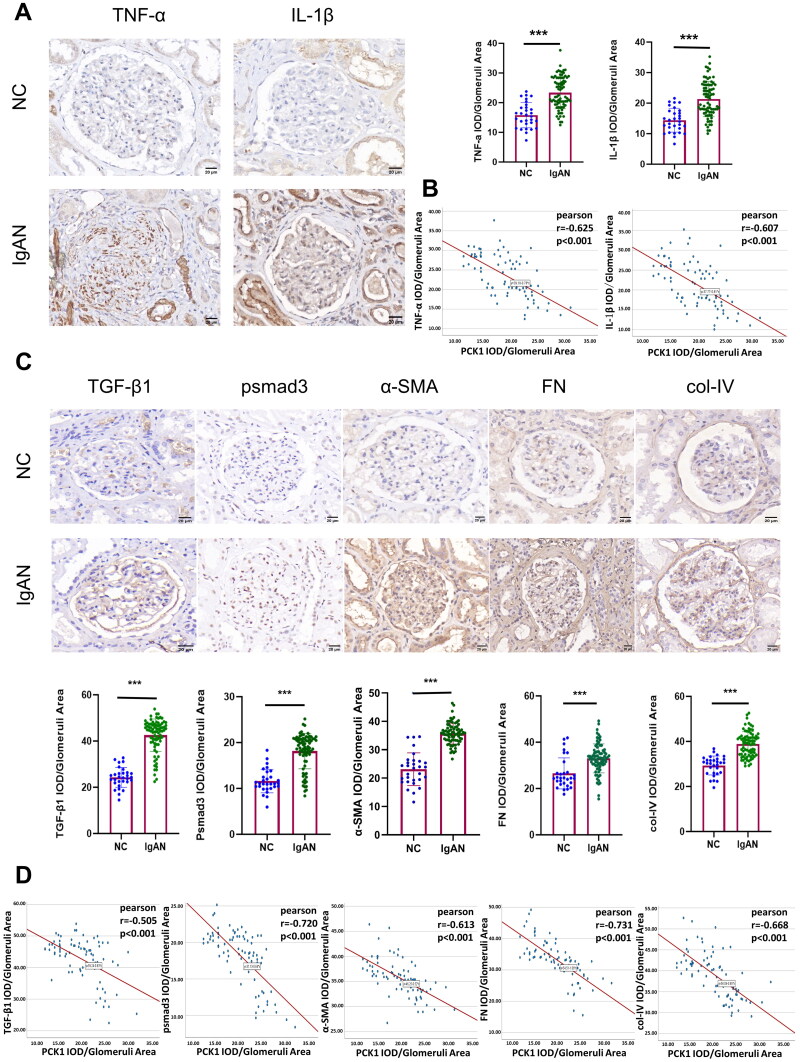
The expression of PCK1 in renal tissue exhibited a negative correlation with the expression of inflammatory chemokines and fibrosis-related markers. Immunohistochemistry and quantification of TNF-α and IL-1β in human renal tissue (patients with IgAN, n = 79 and NC, n = 30). (B) Pearson correlation analysis of the relationship between PCK1 and TNF-α/IL-1β expression. Decreased PCK1 expression was linearly negatively correlated with TNF-α/IL-1β levels (r = -0.625/r = -0.607, *p* < 0.001); (C) Immunohistochemistry and quantification of TGF-β1, psmad3, α-SMA, FN, and col-IV in human renal tissue (patients with IgAN, n = 79 and NC, n = 30). (D) Pearson correlation analysis of the relationship between PCK1 and TGF-β1/psmad3/α-SMA/FN/col-IV expression. Decreased PCK1 expression was linearly negatively correlated with TGF-β1/psmad3/α-SMA/FN/col-IV levels (r = -0.505/r = -0.720/r = -0.613/r = -0.731/r = -0.668). Abbreviations: NC, normal control; TNF-α, tumor necrosis factor α; IL-1β, interleukin 1β; TGF-β1, transforming growth factor-β1; psmad3, Phosphorylated-smad3; α-SMA, α-smooth muscle actin; FN, fibronectin; col-IV, collagen-IV. ***p < 0.001.

Immunohistochemical analysis demonstrated a significant increase in the expression of the inflammatory cytokines TNF-ɑ and IL-1β in renal tissue obtained from patients diagnosed with IgAN compared to normal tissue (Figure 6A). Furthermore, a significant linear inverse relationship was observed between the expression of PCK1 and that of TNF-α (r = −0.625, *p* < 0.001), as well as the expression of IL-1β (r = −0.607, *p* < 0.001) (Figure 6B). Immunohistochemical analysis further demonstrated that in the renal tissues of patients with IgA nephropathy (IgAN), several fibrosis-related markers, including TGF-β1, p-Smad3, α-SMA, FN, and collagen IV, were significantly upregulated (Figure 6C). Additionally, the expression levels of PCK1 exhibited a significant negative correlation with these fibrosis markers: TGF-β1 (r = −0.505, *p* < 0.001), p-Smad3 (r = −0.720, *p* < 0.001), α-SMA (r = −0.613, *p* < 0.001), FN (r = −0.731, *p* < 0.001), and collagen IV (r = −0.668, *p* < 0.001) (Figure 6D).

### Inflammation and fibrosis markers expression differ significantly between NC and pIgA groups in MCs

#### Screening of time and concentration for pIgA1 stimulation on MCs

**Figure 7.**
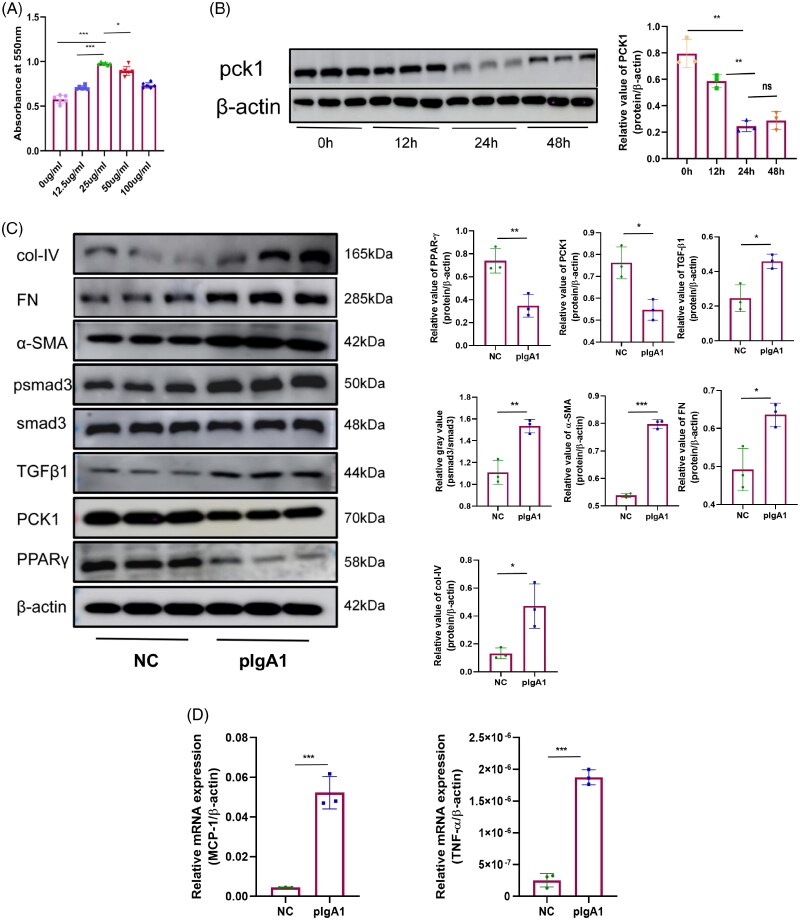
PCK1, inflammatory chemokines, and fibrotic markers expression levels differ significantly between NC and pIgA groups in MCs. (A) Determination of the optimal pIgA1 stimulation concentration in MCs using the MTT assay (24 h); (B) Western blotting analysis of PCK1 protein expression at various time points in MCs induced by pIgA1 (25μg/ml); (C) Western blotting and densitometry analysis of PCK1, PPARγ, TGFβ1, psmad3, α-SMA, FN, and col-IV proteins in NC and pIgA1 groups (n = 3); (D) qRT-PCR analysis of MCP-1 and TNF-α mRNA levels in NC and pIgA1 groups (n = 3). Abbreviations: MCs, mesangial cells; MCP-1, monocyte chemoattractant protein-1; ns, no significant, *p < 0.05, **p < 0.01, ***p < 0.001.

The OD values obtained *via* the MMT method, assessing MCs stimulation with varying pIgA1 concentrations. Results show that 25 μg/mL pIgA1 significantly promoted cell proliferation compared to other concentrations (Figure 7A). Different time points were selected for cell stimulation, and Western blotting analysis was used to assess PCK1 protein expression. A significant decrease in PCK1 expression was observed at 24 h (Figure 7B). Consequently, the IgAN mesangial cell disease model was established by stimulating cells with a concentration of 25 μg/ml pIgA1 for a duration of 24 h.

#### PCK1 expression is decreased in pIgA1-stimulated MCs

Western blotting analysis revealed significantly decreased protein expression levels of PCK1 and PPARγ in the pIgA1 group compared to the NC group. Conversely, the expression levels of fibrosis markers TGFβ1, psmad3, α-SMA, FN, and col-IV were notably increased (Figure 7C). Additionally, the pIgA1 group exhibited a noteworthy elevation in the mRNA expression levels of inflammation markers MCP-1 and TNF-α, as demonstrated by qRT-PCR analysis (Figure 7D).

### PCK1 overexpression decreases inflammatory and fibrotic factors in MCs

**Figure 8.**
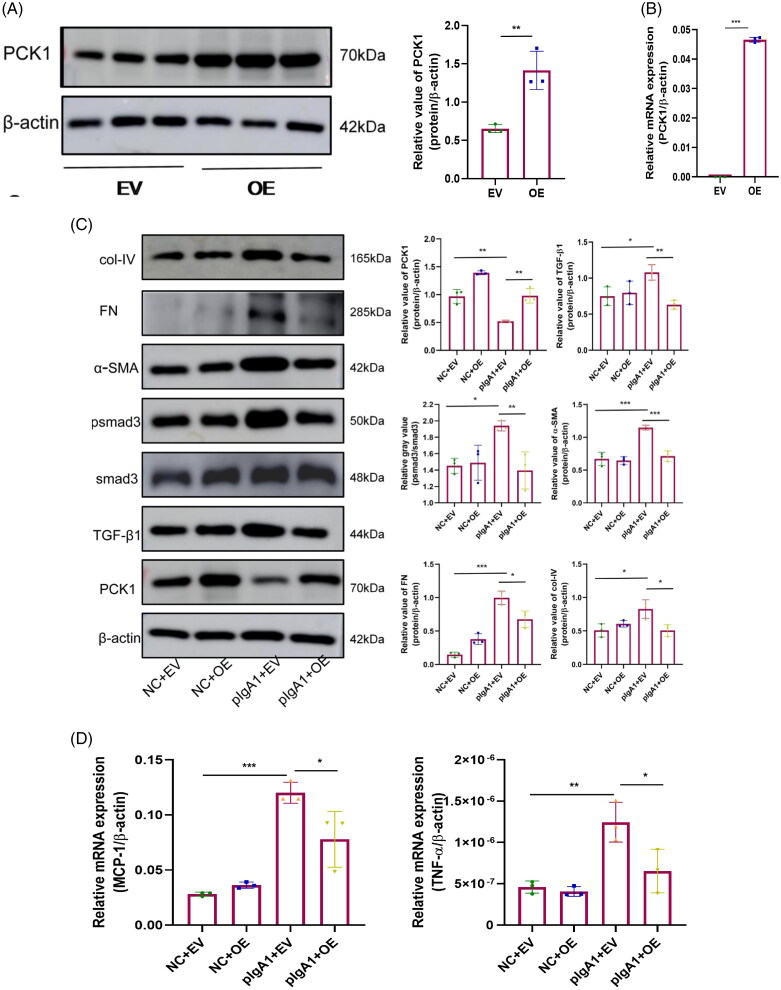
Impact of PCK1 gene overexpression on inflammatory and fibrotic factors in MCs in the pIgA group. Western blotting and densitometry analyses of PCK1 protein in MCs transfected with either empty vector or PCK1 overexpression plasmids (n = 3). (B) qRT-PCR analysis of PCK1 mRNA in SV40 cells transfected with either empty vector or PCK1 overexpression plasmids (n = 3). (C) Western blotting and densitometry analyses of PCK1, PPARγ, TGFβ1, psmad3, α-SMA, FN, and col-IV proteins in SV40 cells from the natural control group and after pIgA1 stimulation, transfected with either empty vector or PCK1 overexpression plasmids (n = 3). (D) qRT-PCR analysis of MCP-1 and TNF-α mRNA in NC and pIgA1 groups transfected with either empty vector or PCK1 overexpression plasmids (n = 3). Abbreviations: MCs, mesangial cells; EV, empty vector; OE, overexpression. *p < 0.05, **p < 0.01, ***p < 0.001.

After transfection with the PCK1 overexpression lentivirus, Western blotting and RT-qPCR confirmed the upregulation of PCK1. The results showed a notable rise in the levels of PCK1 mRNA and protein expression in MCs transfected with the PCK1 overexpression lentivirus (OE group) compared to the EV group (Figure 8A,B). Following PCK1 overexpression, the TGFβ1 signaling pathway in the disease model group was significantly suppressed, with marked reductions in the protein expression levels of fibrosis markers such as α-SMA, FN, and col-IV (Figure 8C). Furthermore, the qRT-PCR analysis demonstrated a significant reduction in the mRNA expression levels of MCP-1 and TNF-α, which are inflammatory markers, in the pIgA1 + OE group compared to those observed in the pIgA1 + EV group (Figure 8D).

### The protective effects of PCK1 overexpression and rosiglitazone on cell fibrosis in MCs are comparable

**Figure 9.**
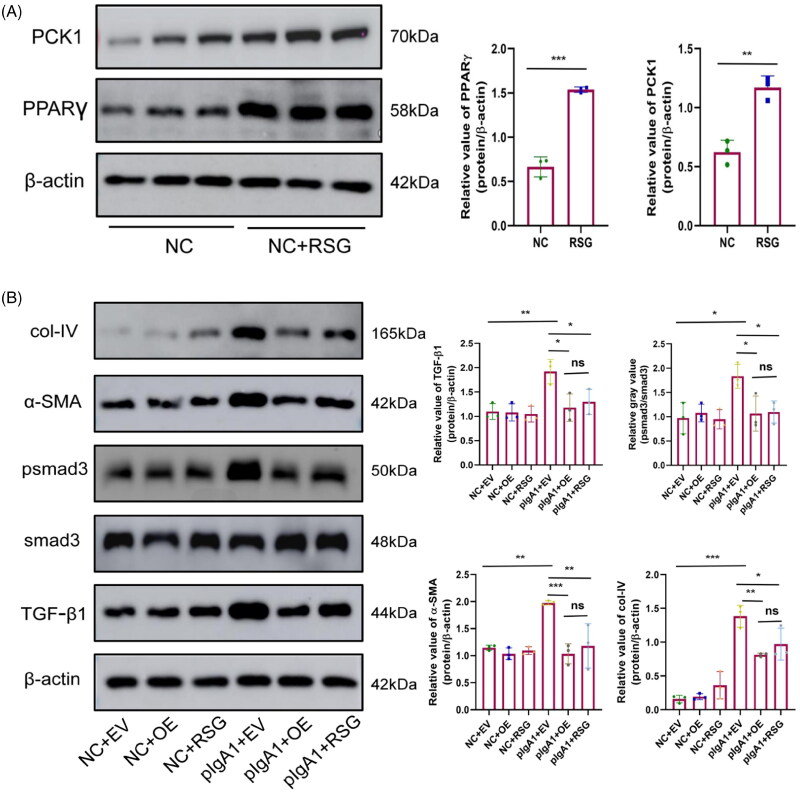
Comparison of the impact of PCK1 overexpression and rosiglitazone on cellular fibrosis. Western blotting and densitometry analyses of PPARγ protein in MCs treated with RSG (10 μM, 48 h); (B) Western blotting and densitometry analyses of TGFβ1, psmad3, α-SMA, and col-IV proteins in SV40 cells from the natural control group and after pIgA1 stimulation, transfected with either empty vector or PCK1 overexpression plasmids, and treated with RSG (n = 3). Abbreviations: MCs, mesangial cells; RSG, rosiglitazone; EV, empty vector; OE, overexpression; ns, no significant. *p < 0.05, **p < 0.01, ***p < 0.001.

MCs were treated with 10 μM rosiglitazone for 48 h, leading to a significant upregulation of both PPARγ and PCK1 protein expression levels. (Figure 9A). Western blotting analysis showed that the TGFβ1 signaling pathway in the pIgA1 + EV group was significantly upregulated compared to the NC+EV group, along with a notable increase in the expression of fibrosis markers α-SMA and col-IV proteins. However, after PCK1 overexpression or rosiglitazone treatment, the TGFβ1 signaling pathway in the disease group was significantly suppressed compared to the pIgA1 + EV group, with a marked decrease in the expression of α-SMA and col-IV proteins. There was negligible disparity observed between the two groups (Figure 9B).

## Discussion

The incidence of IgAN is notably high. Despite its relatively slow progression, it remains a significant contributor to end-stage renal disease. The current ‘gold standard’ for diagnosing and monitoring IgAN remains invasive renal tissue biopsy, and there is a paucity of biomarkers to track disease progression or evaluate treatment response [33]. Consequently, it is imperative to investigate new clinical markers and therapeutic targets. In order to identify genes and signaling pathways closely associated with IgAN clinical traits, a comprehensive analysis of GEO datasets was performed in this study. Through initial screening of the GSE104948 and GSE37460 datasets, a total of 56 common DEGs were identified. KEGG enrichment analysis revealed that four differential genes were enriched in the PPARγ pathway, with PCK1 identified as the most strongly associated downregulated gene. The downregulation of this gene in the disease group was statistically significant in both datasets. ROC curve analysis of mRNA data showed its potential as a reliable diagnostic marker for IgAN. Validation dataset GSE93798 confirmed the conclusion. We identified proteins associated with PCK1 through the STRING database (version 12.0) and constructed a protein-protein interaction network. Following enrichment analysis, we found that PCK1 was significantly enriched in pathways related to AMPK and PPARγ signaling.

A substantial body of evidence confirms that the PPAR signaling pathway is critically involved in the pathogenesis and progression of kidney diseases, and PPARγ agonists exhibit beneficial therapeutic effects in diabetic nephropathy and other renal disorders. In streptozotocin (STZ)-induced type 1 diabetic rodents, PPARγ activation has been shown to ameliorate proteinuria independently of blood glucose regulation [34]. In polycystic kidney disease models, short-term administration of rosiglitazone can delay cyst progression and preserve renal function. Moreover, long-term use of this drug has been associated with extended lifespan in these animals. The underlying mechanisms may involve inhibition of inflammation and fibrosis by activating the PPARγ signaling pathway [35]. Additionally, PPARγ activation has been reported to protect against ischemia/reperfusion (I/R) injury in the kidneys [36]. Several reviews have highlighted its effects on renal inflammation and fibrosis [37], elucidating its impact on inflammatory factors such as TNF-α, IL-1β, IL-6, and the TGF-β-Smad pathway, which contribute to fibrosis during kidney disease progression. Its role in IgA nephropathy (IgAN) has also been documented, including its influence on renal cell ferroptosis and chronic fibrosis following overexpression [38,39]. Therefore, we selected the key downstream gene PCK1 in the PPAR signaling pathway as our research focus to investigate its role in the pathogenesis and progression of IgAN.

We performed immunohistochemical staining on the renal tissue specimens of 79 patients with IgAN who underwent renal biopsy at the First Affiliated Hospital of Anhui Medical University. Compared to the normal control group, both PCK1 and PPARγ expression were lower in patients with IgAN. Additionally, using different pathological classification methods such as Katafuchi and Oxford classification, a correlation was observed between the severity of glomerular lesions in patients with IgAN and the expression of PCK1 in the glomeruli. In the immunohistochemical results of pathological specimens from patients with IgAN, a negative correlation was observed between PCK1 expression and clinical parameters such as 24-h proteinuria, urine protein creatinine ratio, serum creatinine, and urea nitrogen. Conversely, a positive correlation was observed between factors such as glomerular filtration rate and serum albumin levels. The aforementioned experimental results demonstrate that, both at the pathological and clinical manifestation levels, a decrease in this indicator exhibits significant predictive value for disease progression. Specifically, lower expression of this protein in the glomerulus correlate with more severe disease progression. Consequently, we propose that reduced expression of glomerular PCK1 protein serves as a critical predictor of IgAN progression. Similar conclusions were also made by the team of Sophie De Seigneux from the University of Geneva in Switzerland, who published in JASN that the downregulation of PCK1 could predict the progression of chronic fibrotic lesions in CKD [40]. However, our verification in IgAN demonstrated that the clinical and pathological severity of the disease is closely associated with this gene. Consequently, we propose that reduced expression of the PCK1 protein may serve as a potential predictor of prognosis in IgAN.

The abnormal proliferation of mesangial cells and the increased synthesis of extracellular matrix are considered the most critical pathological features of IgAN. The immunofluorescence staining of our previously examined renal pathological specimens demonstrated that PCK1 exhibited significant co-localization with the mesangial cell-specific marker PDGFR-β. This suggests that PCK1 may play an important role in mesangial cells. Consequently, we selected the SV40-transformed mouse mesangial cell line for our experiments. Our data showed that PCK1 and PPARγ were significantly downregulated in the pIgA1 group compared to the control group. Additionally, we observed activation of the TGF-β1/Smad3 signaling pathway, upregulation of FN and Col-IV, as well as increased levels of inflammatory cytokines such as MCP-1 and TNF-α. These results suggest that in MCs of the IgAN model, there is a significant reduction in PCK1 and PPARγ expression, accompanied by elevated expression of inflammatory factors, extracellular matrix proteins, and fibrotic growth factors. But both inflammatory factors and extracellular matrix/fibrosis markers were significantly inhibited in the IgAN MCs model with PCK1 overexpression. This suggests that PCK1 overexpression exerts a protective effect against inflammation and fibrosis in mesangial cells.

Currently, several studies have demonstrated that PPARγ plays a pivotal role in the pathophysiology of IgAN. The PPARγ agonist rosiglitazone has been shown to inhibit mesangial cell proliferation, serine protease activity, and TGFβ expression, while activating the TGFβ-Smad3 signaling pathway, thereby preventing the progression of glomerular sclerosis [41]. Given that PCK1 is a downstream gene of the PPARγ signaling pathway, would its overexpression exert similar protective effects as rosiglitazone? In our experiment, we observed that rosiglitazone stimulation led to increased protein expression levels of PPARγ and PCK1. Notably, both overexpression of PCK1 alone and rosiglitazone treatment similarly inhibited the TGFβ1-Smad3 pathway as well as the expression of αSMA and Col-IV in Mcs from the IgAN model, with no statistically significant differences between the two approaches. Therefore, we speculated that the effect of PPARγ agonists on mitigating chronic fibrotic changes in mesangial cells may be mediated through upregulation of PCK1. However, this hypothesis requires further validation. But at this stage, our findings indicated that overexpression of PCK1 exerts an inhibitory effect on chronic mesangial cell fibrosis in the IgAN model, similar to that observed with rosiglitazone. This observation may offer novel therapeutic insights for the treatment of IgAN.

## Conclusion

Our research demonstrated that in the glomeruli of IgAN patients, the expression of PCK1, a key gene in the PPARγ signaling pathway, was significantly reduced and correlates strongly with both clinical progression and pathological alterations. The overexpression of PCK1 ­significantly alleviated pIgA1-induced inflammation and fibrosis in mesangial cells. These findings suggested that PCK1 may serve as a reliable predictor of IgAN and it could potentially represent a novel therapeutic target.

## Supplementary Material

Supplementary table 4.docx

Supplementary table 1.docx

Supplementary table 5.docx

Supplementary table 2.docx

Supplementary table 3.docx

## Data Availability

The datasets produced and/or examined in the present investigation can be obtained by contacting the corresponding author through a reasonable inquiry.

## References

[1] Coppo R. IgA nephropathy: a European perspective in the corticosteroid treatment. Kidney Dis (Basel). 2018;4(2):58–64. doi: 10.1159/000487265. 29998120 PMC6029231

[2] Lai KN, Tang SCW, Schena FP, et al. IgA nephropathy. Nat Rev Dis Primers. 2016;2(1):16001. doi: 10.1038/nrdp.2016.1. 27189177

[3] Barbour SJ, Cattran DC, Kim SJ, et al. Individuals of Pacific Asian origin with IgA nephropathy have an increased risk of progression to end-stage renal disease. Kidney Int. 2013;84(5):1017–1024. 23739233 10.1038/ki.2013.210

[4] Tam FWK, Pusey CD. TESTING corticosteroids in IgA nephropathy: a continuing challenge. Clin J Am Soc Nephrol. 2018;13(1):158–160. 29237704 10.2215/CJN.10560917 PMC5753322

[5] Zhang H, Barratt J. Is IgA nephropathy the same disease in different parts of the world? Semin Immunopathol. 2021;43(5):707–715. doi: 10.1007/s00281-021-00884-7. 34417628

[6] Roberts IS. Pathology of IgA nephropathy. Nat Rev Nephrol. 2014;10(8):445–454. doi: 10.1038/nrneph.2014.92. 24861083

[7] Schena FP, Serino G, Sallustio F, et al. Omics studies for comprehensive understanding of immunoglobulin A nephropathy: state-of-the-art and future directions. Nephrol Dial Transplant. 2018;33(12):2101–2112. 29905852 10.1093/ndt/gfy130

[8] Maixnerova D, Reily C, Bian Q, et al. Markers for the progression of IgA nephropathy. J Nephrol. 2016;29(4):535–541. doi: 10.1007/s40620-016-0299-0. 27142988 PMC5548426

[9] Barbour SJ, Espino-Hernandez G, Reich HN, et al. The MEST score provides earlier risk prediction in lgA nephropathy. Kidney Int. 2016;89(1):167–175. doi: 10.1038/ki.2015.322. 26759049

[10] Rovin BH, Adler SG, Barratt J, et al. Executive summary of the KDIGO 2021 Guideline for the Management of Glomerular Diseases. Kidney Int. 2021;100(4):753–779. 34556300 10.1016/j.kint.2021.05.015

[11] Zhu L, Yin Q, Irwin DM, et al. Phosphoenolpyruvate carboxykinase 1 gene (Pck1) displays parallel evolution between Old World and New World fruit bats. PLoS One. 2015;10(3):e0118666. doi: 10.1371/journal.pone.0118666. 25807515 PMC4373879

[12] Yu S, Meng S, Xiang M, et al. Phosphoenolpyruvate carboxykinase in cell metabolism: roles and mechanisms beyond gluconeogenesis. Mol Metab. 2021;53:101257. doi: 10.1016/j.molmet.2021.101257. 34020084 PMC8190478

[13] Xu D, Wang Z, Xia Y, et al. The gluconeogenic enzyme PCK1 phosphorylates INSIG1/2 for lipogenesis. Nature. 2020;580(7804):530–535. doi: 10.1038/s41586-020-2183-2. 32322062

[14] Ko CW, Counihan D, Wu J, et al. Macrophages with a deletion of the phosphoenolpyruvate carboxykinase 1 (Pck1) gene have a more proinflammatory phenotype. J Biol Chem. 2018;293(9):3399–3409. 29317502 10.1074/jbc.M117.819136 PMC5836109

[15] Ye Q, Liu Y, Zhang G, et al. Deficiency of gluconeogenic enzyme PCK1 promotes metabolic-associated fatty liver disease through PI3K/AKT/PDGF axis activation in male mice. Nat Commun. 2023;14(1):1402. doi: 10.1038/s41467-023-37142-3. 36918564 PMC10015095

[16] Xiang J, Chen C, Liu R, et al. Gluconeogenic enzyme PCK1 deficiency promotes CHK2 O-GlcNAcylation and hepatocellular carcinoma growth upon glucose deprivation. J Clin Invest. 2021;131(8):e144703. 33690219 10.1172/JCI144703 PMC8262473

[17] Shi L, An S, Liu Y, et al. PCK1 regulates glycolysis and tumor progression in clear cell renal cell carcinoma through LDHA. Onco Targets Ther. 2020;13:2613–2627. doi: 10.2147/OTT.S241717. 32280238 PMC7125947

[18] Tuo L, Xiang J, Pan X, et al. PCK1 negatively regulates cell cycle progression and hepatoma cell proliferation via the AMPK/p27Kip1 axis. J Exp Clin Cancer Res. 2019;38(1):50. doi: 10.1186/s13046-019-1029-y. 30717766 PMC6360696

[19] Verissimo T, Dalga D, Arnoux G, et al. PCK1 is a key regulator of metabolic and mitochondrial functions in renal tubular cells. Am J Physiol Renal Physiol. 2023;324(6):F532–F543. 37102687 10.1152/ajprenal.00038.2023 PMC10202477

[20] Hasegawa K, Sakamaki Y, Tamaki M, et al. PCK1 protects against mitoribosomal defects in diabetic nephropathy in mouse models. J Am Soc Nephrol. 2023; Aug 134(8):1343–1365. doi: 10.1681/ASN.0000000000000156. 37199399 PMC10400109

[21] Liu P, Lassén E, Nair V, et al. Transcriptomic and proteomic profiling provides insight into mesangial cell function in IgA nephropathy. J Am Soc Nephrol. 2017;28(10):2961–2972. 28646076 10.1681/ASN.2016101103 PMC5619958

[22] Berthier CC, Bethunaickan R, Gonzalez-Rivera T, et al. Cross-species transcriptional network analysis defines shared inflammatory responses in murine and human lupus nephritis. J Immunol. 2012;189(2):988–1001. doi: 10.4049/jimmunol.1103031. 22723521 PMC3392438

[23] Grayson PC, Eddy S, Taroni JN, et al. Metabolic pathways and immunometabolism in rare kidney diseases. Ann Rheum Dis. 2018;77(8):1226–1233. 29724730 10.1136/annrheumdis-2017-212935 PMC6045442

[24] Li Y, Xia M, Peng L, et al. Downregulation of miR-214-3p attenuates mesangial hypercellularity by targeting PTEN-mediated JNK/c-Jun signaling in IgA nephropathy. Int J Biol Sci. 2021;17(13):3343–3355. doi: 10.7150/ijbs.61274. 34512151 PMC8416718

[25] Hyun YY, Kim IO, Kim MH, et al. Adipose-derived stem cells improve renal function in a mouse model of IgA nephropathy. Cell Transplant. 2012;21(11):2425–2439. doi: 10.3727/096368912X639008. 22525004

[26] Xia M, Liu D, Tang X, et al. Dihydroartemisinin inhibits the proliferation of IgAN mesangial cells through the mTOR signaling pathway. Int Immunopharmacol. 2020; Mar80:106125. 31931362 10.1016/j.intimp.2019.106125

[27] Kumar N, Dey CS. Development of insulin resistance and reversal by thiazolidinediones in C2C12 skeletal muscle cells. Biochem Pharmacol. 2003;65(2):249–257. doi: 10.1016/s0006-2952(02)01509-5. 12504800

[28] Sander TL, Noll L, Klinkner DB, et al. Rosiglitazone antagonizes vascular endothelial growth factor signaling and nuclear factor of activated T cells activation in cardiac valve endothelium. Endothelium. 2006;13(3):181–190. doi: 10.1080/10623320600760308. 16840174

[29] Zhao D, Guo J, Liu L, et al. Rosiglitazone attenuates high glucose-induced proliferation, inflammation, oxidative stress and extracellular matrix accumulation in mouse ­mesangial cells through the Gm26917/miR-185-5p pathway. Endocr J. 2021;68(7):751–762. doi: 10.1507/endocrj.EJ20-0783. 33790061

[30] Lennon R, Welsh GI, Singh A, et al. Rosiglitazone enhances glucose uptake in glomerular podocytes using the glucose transporter GLUT1. Diabetologia. 2009;52(9):1944–1952. doi: 10.1007/s00125-009-1423-7. 19533082 PMC7614273

[31] Deng W, Wei X, Dong Z, et al. Identification of fibroblast activation-related genes in two acute kidney injury models. PeerJ. 2021;9:e10926. doi: 10.7717/peerj.10926. 33777519 PMC7982076

[32] Liu R, Gou D, Xiang J, et al. O-GlcNAc modified-TIP60/KAT5 is required for PCK1 deficiency-induced HCC metastasis. Oncogene. 2021;40(50):6707–6719. doi: 10.1038/s41388-021-02058-z. 34650217 PMC8677624

[33] Al-Lawati AI, Reich HN. Is there a role for immunosuppression in immunoglobulin A nephropathy? Nephrol Dial Transplant. 2017;32(suppl_1):i30–i36. doi: 10.1093/ndt/gfw342. 28391341

[34] Flaquer M, Lloberas N, Franquesa M, et al. The combination of sirolimus and rosiglitazone produces a renoprotective effect on diabetic kidney disease in rats. Life Sci. 2010;87(5-6):147–153. doi: 10.1016/j.lfs.2010.06.004. 20600147

[35] Dai B, Liu Y, Mei C, et al. Rosiglitazone attenuates development of polycystic kidney disease and prolongs survival in Han: SPRD rats. Clin Sci (Lond). 2010;119(8):323–333. doi: 10.1042/CS20100113. 20507283

[36] ] Collino M, Patel NSA, Lawrence KM, et al. The selective PPARgamma antagonist GW9662 reverses the protection of LPS in a model of renal ischemia-reperfusion. Kidney Int. 2005;68(2):529–536. doi: 10.1111/j.1523-1755.2005.00430.x. 16014029

[37] Tovar-Palacio C, Torres N, Diaz-Villaseñor A, et al. The role of nuclear receptors in the kidney in obesity and metabolic syndrome. Genes Nutr. 2012;7(4):483–498. Epub 2012 Apr 25. PMID: 22532116; PMCID: PMC3448033. doi: 10.1007/s12263-012-0295-5. 22532116 PMC3448033

[38] Chan WL, Leung JC, Chan LY, et al. BMP-7 protects mesangial cells from injury by polymeric IgA. Kidney Int. 2008;74(8):1026–1039. 18496506 10.1038/ki.2008.209

[39] Wu J, Shao X, Shen J, et al. Downregulation of PPARα mediates FABP1 expression, contributing to IgA nephropathy by stimulating ferroptosis in human mesangial cells. Int J Biol Sci. 2022;18(14):5438–5458. doi: 10.7150/ijbs.74675. 36147466 PMC9461665

[40] Verissimo T, Faivre A, Rinaldi A, et al. Decreased renal gluconeogenesis is a hallmark of chronic kidney disease. J Am Soc Nephrol. 2022;33(4):810–827. 35273087 10.1681/ASN.2021050680 PMC8970457

[41] Ouyang Y, Xie J, Yang M, et al. Underweight is an independent risk factor for renal function deterioration in patients with IgA nephropathy. PLoS One. 2016;11(9):e0162044. doi: 10.1371/journal.pone.0162044. 27611091 PMC5017745

