# Supplementary material for: Protective effect of phosphoenolpyruvate carboxykinase 1 on inflammation and fibrotic progression of IgA nephropathy
Source: Ren Fail. 2025 May 29;47(1):2508297. doi: 10.1080/0886022X.2025.2508297 (PMC12128133; doi:10.1080/0886022X.2025.2508297)
Supplement: Supplementary table 4.docx [file IRNF_A_2508297_SM3115.docx]

Supplementary table 4. primers of PCK1,TNF-α, IL-1β, MCP-1, and β-actin

| Primer | Forward primer | Reverse primer |
| --- | --- | --- |
| PCK1 | AAAACGGCCTGAACCTCTCG | ACACAGCTCAGCGTTATTCTC |
| MCP-1 | CAAACTGAAGCTCGCACTCTCGCC | ATTCTTGGGTTCTGGAGTGAGTGTTCA |
| IL-1β | CCACAGACCTTCCAGGAGAATG | GTGCAGTTCAGTGATCGTACAGG |
| TNF-a | CTCTTCTGCCTGCTGCACTTTG | ATGGGCTACAGGCTTGTCACTC |
| β-actin | AGGCCAACCGTGAAAAGATG | TGGCGTGAGGGAGAGCATAG |

**Abbreviations:** PCK1: Phosphoenolpyruvate carboxykinase 1; MCP-1: Monocyte chemoattractant protein-1; IL-1β: Interleukin-1β; TNF-α: Tumor Necrosis Factor-alpha.
