# Supplementary material for: Protective effect of phosphoenolpyruvate carboxykinase 1 on inflammation and fibrotic progression of IgA nephropathy
Source: Ren Fail. 2025 May 29;47(1):2508297. doi: 10.1080/0886022X.2025.2508297 (PMC12128133; doi:10.1080/0886022X.2025.2508297)
Supplement: Supplementary table 1.docx [file IRNF_A_2508297_SM3112.docx]

Supplementary table 1. Basic clinical data of patients with IgAN and healthy controls

| clinical indicators | IgAN(n=79) | NC(n=30) | Normal ranges |
| --- | --- | --- | --- |
| Gender male/female | 40/39 | 9/21 | - |
| Age (years)  [M (P25,P75)] | 41.00 (30.00,51.00) | 49.00 (42.00,55.00) | - |
| MAP (mmHg)  [Means ±SD] | 100.86±13.95 | 105.20±12.06 | 70.00-100.00 |
| FBG (mmol/l)  [Means ±SD] | 4.57±0.73 | 4.51±0.67 | 3.89-6.11 |
| Albumin (g/l)  [Means ±SD] | 40.94±5.29 | 41.90±3.30 | 40.00-55.00 |
| Serum creatinine (umol/l)  [M (P25,P75)] | 104.28 (64.00,127.00) | 62.10 (55.40,72.60) | 57.00-97.00 |
| BUN (mmol/l)  [M (P25,P75)] | 6.53 (4.84,7.71) | 5.56 (4.98,6.28) | 3.10-8.00 |
| Uric acid (mmol/l)  [Means ±SD] | 384.14±117.3 | 347.17±72.26 | 208.00-428.00 |
| eGFR (mL/min·1.73 m^2^)  [M (P25,P75)] | 87.37 (60.00,113.00) | 121.00 (108.00,129.00) | >90.00 |
| Cholesterol (mmol/l)  [M (P25,P75)] | 4.75 (3.98,5.28) | 4.19 (3.98,4.81) | 2.86-5.98 |
| Triglyceride (mmol/l)  [M (P25,P75)] | 1.87 (1.12,2.26) | 1.44 (1.00,1.79) | 0.56-1.70 |
| 24U-pro (g/24h)  [M (P25,P75)] | 1.64 (0.52,1.77) | 0.018 (0.015,0.026) | 0.00-0.15 |
| mAlb/U-CRE (mg/g)  [M (P25,P75)] | 1384.72 (412.00,1795.00) | 21.00 (16.00,26.00) | 0-30.00 |
| Serum immunoglobulin A (g/l)  [M (P25,P75)] | 3.05 (2.15,3.66) | - | 0.70-4.00 |
| Serum complement 3 (g/l)  [M (P25,P75)] | 1.18 (1.04,1.30) | - | 0.90-1.80 |

**Abbreviations：**NC: negative control; IgAN: immunoglobulin A nephropathy; MAP: mean arterial pressure; FBG: fasting blood-glucose; BUN: blood urea nitrogen; eGFR: estimated glomerular filtration rate; 24U-pro: 24h urine protein; mAlb/U-CRE: urinary microalbumin-to-creatinine ratio.
