# Supplementary material for: Protective effect of phosphoenolpyruvate carboxykinase 1 on inflammation and fibrotic progression of IgA nephropathy
Source: Ren Fail. 2025 May 29;47(1):2508297. doi: 10.1080/0886022X.2025.2508297 (PMC12128133; doi:10.1080/0886022X.2025.2508297)
Supplement: Supplementary table 5.docx [file IRNF_A_2508297_SM3106.docx]

Supplementary table 5. Correlation analysis between PCK1 level and clinicopathological indicator

| clinical indicators | IgAN (n=79) | Correlation Coefficient (*r*) | *P* value |
| --- | --- | --- | --- |
| MAP (mmHg)  [Means ±SD] | 100.86±13.95 | -0.167 | 0.142 |
| FBG (mmol/l)  [Means ±SD] | 4.57±0.73 | 0.113 | 0.321 |
| Albumin (g/l)  [Means ±SD] | 40.94±5.29 | 0.483 | <0.001*** |
| Serum creatinine (umol/l)  [M (P25,P75)] | 104.28 (64.00,127.00) | -0.421 | <0.001*** |
| BUN (mmol/l)  [M (P25,P75)] | 6.53 (4.84,7.71) | -0.296 | 0.008** |
| Uric acid (mmol/l)  [Means ±SD] | 384.14±117.3 | -0.163 | 0.15 |
| eGFR (mL/min·1.73 m^2^)  [M (P25,P75)] | 87.37 (60.00,113.00) | 0.339 | 0.002** |
| 24U-pro (g/24h)  [M (P25,P75)] | 1.64 (0.52,1.77) | -0.927 | <0.001*** |
| mAlb/U-CRE (mg/g)  [M (P25,P75)] | 1384.72 (412.00,1795.00) | -0.81 | <0.001*** |
| Serum immunoglobulin A (g/l)  [M (P25,P75)] | 3.05 (2.15,3.66) | 0.127 | 0.264 |
| Complement 3 (g/l)  [M (P25,P75)] | 1.18 (1.04,1.30) | 0.066 | 0.565 |

*Significant level at P ≤ 0.05, **Significant level at P ≤ 0.01 and *** significant level at P ≤ 0.001.

**Abbreviations：**IgAN: immunoglobulin A nephropathy; MAP: mean arterial pressure; FBG: fasting blood-glucose; BUN: blood urea nitrogen; eGFR: estimated glomerular filtration rate; 24U-pro: 24h urine protein; mAlb/U-CRE: Urinary microalbumin-to-creatinine ratio.
