# Supplementary material for: Protective effect of phosphoenolpyruvate carboxykinase 1 on inflammation and fibrotic progression of IgA nephropathy
Source: Ren Fail. 2025 May 29;47(1):2508297. doi: 10.1080/0886022X.2025.2508297 (PMC12128133; doi:10.1080/0886022X.2025.2508297)
Supplement: Supplementary table 2.docx [file IRNF_A_2508297_SM3101.docx]

Supplementary table 2. Basic histological features of patients with IgAN

| Oxford classfication (MEST-C) | |
| --- | --- |
| M0/M1 | 22/57 |
| E0/E1 | 51/28 |
| S0/S1 | 38/41 |
| T0/T1-2 | 54/25 |
| C0/C1-2 | 52/27 |
| Katafuchi semi-quantitative criteria [M(P25,P75)] | |
| Glomerulus scores | 5.00(3.00,6.00) |
| Tubulointerstitial scores | 3.00(3.00,6.00) |
| Vessel scores | 3.00(2.00,3.00) |

**Abbreviations:** M: mesangial hypercellularity; E:endocapillary hypercellularity; S: segmental glomerulosclerosis; T: tubular atrophy/interstitial fibrosis; C: crescents.
