# Supplementary material for: Protective effect of phosphoenolpyruvate carboxykinase 1 on inflammation and fibrotic progression of IgA nephropathy
Source: Ren Fail. 2025 May 29;47(1):2508297. doi: 10.1080/0886022X.2025.2508297 (PMC12128133; doi:10.1080/0886022X.2025.2508297)
Supplement: Supplementary table 3.docx [file IRNF_A_2508297_SM3092.docx]

Supplementary table 3. Primer sequence

| Primer | Forward primer | Reverse primer |
| --- | --- | --- |
| Meth-PCK1 | TCGGTTATATTTTTAAGGAAAACGT | CCTAATCCTCCAAATACCTATCGAT |
| Unmeth-PCK1 | TTGGTTATATTTTTAAGGAAAATGT | CTAATCCTCCAAATACCTATCAAT |

**Abbreviations:** PCK1: Phosphoenolpyruvate carboxykinase 1.
